# Caregivers experiences in the WeChat groups of the WHO iSupport online course in China

**DOI:** 10.1002/alz70858_102983

**Published:** 2025-12-25

**Authors:** Mengmeng Xia, Mei Zhao, Haifeng Zhang, Huali Wang

**Affiliations:** ^1^ Beijing Jiahua Social Work Center, Beijing, Beijing, China; ^2^ Peking University Institute of Mental Health (Sixth Hospital), Beijing, Beijing, China; ^3^ Beijing Haidian Psychological Rehabilitation Hospital, Beijing, Beijing, China; ^4^ Dementia Care and Research Center, Peking University Institute of Mental Health (Sixth Hospital), Beijing, China; ^5^ NHC Key Laboratory for Mental Disorders, Beijing, Beijing, China; ^6^ National Clinical Research Center for Mental Disorders, Beijing, China

## Abstract

**Background:**

The WHO iSupport for Dementia Program has been disseminated globally in multiple languages, including Chinese. This program is primarily delivered via a web‐based platform, enabling caregivers to plan their study schedules, engage in self‐paced learning, and tailor the iSupport modules to meet their individual needs. The present study aims to explore the perspectives of Chinese dementia caregivers on using the iSupport online program.

**Method:**

Eight out of twenty caregivers enrolled in the iSupport intervention participated in this study. The intervention comprised three components: (1) caregiver self‐learning through the WHO iSupport online course; (2) participation in caregiver support groups; and (3) interaction with other caregivers via a WeChat group. Audio recordings from monthly online meetings were transcribed verbatim for data analysis. Facilitators documented their support activities in monthly portfolios. Thematic analysis was applied to data collected from online caregiver meetings and facilitator portfolios.

**Result:**

Following a 6‐month iSupport intervention, caregivers recognized the importance of validating the experiences of persons living with dementia. They found that iSupport could be adapted to their individual needs and helped them change their communication approaches with dementia patients. Additionally, participants reported that iSupport and WeChat group support enhanced their resilience when living with someone who has dementia and supported them through self‐reflection. Through self‐learning, caregivers also acknowledged gaps in improving care practices and achieving better quality and outcomes.

**Conclusion:**

Our findings indicate that psychoeducation for dementia caregivers through the iSupport online course and WeChat group support is feasible. Combining self‐directed learning with peer interaction may improve caregivers' attitudes and skills related to dementia care.

